# Initiation of male sperm-transfer behavior in *Caenorhabditis elegans *requires input from the ventral nerve cord

**DOI:** 10.1186/1741-7007-4-26

**Published:** 2006-08-15

**Authors:** Gary Schindelman, Allyson J Whittaker, Jian Yuan Thum, Shahla Gharib, Paul W Sternberg

**Affiliations:** 1 Howard Hughes Medical Institute and Division of Biology, California Institute of Technology, Pasadena, CA 91125, USA

## Abstract

**Background:**

The *Caenorhabditis elegans *male exhibits a stereotypic behavioral pattern when attempting to mate. This behavior has been divided into the following steps: response, backing, turning, vulva location, spicule insertion, and sperm transfer. We and others have begun in-depth analyses of all these steps in order to understand how complex behaviors are generated. Here we extend our understanding of the sperm-transfer step of male mating behavior.

**Results:**

Based on observation of wild-type males and on genetic analysis, we have divided the sperm-transfer step of mating behavior into four sub-steps: initiation, release, continued transfer, and cessation. To begin to understand how these sub-steps of sperm transfer are regulated, we screened for ethylmethanesulfonate (EMS)-induced mutations that cause males to transfer sperm aberrantly. We isolated an allele of *unc-18*, a previously reported member of the Sec1/Munc-18 (SM) family of proteins that is necessary for regulated exocytosis in *C. elegans *motor neurons. Our allele, *sy671*, is defective in two distinct sub-steps of sperm transfer: initiation and continued transfer. By a series of transgenic site-of-action experiments, we found that motor neurons in the ventral nerve cord require UNC-18 for the initiation of sperm transfer, and that UNC-18 acts downstream or in parallel to the SPV sensory neurons in this process. In addition to this neuronal requirement, we found that non-neuronal expression of UNC-18, in the male gonad, is necessary for the continuation of sperm transfer.

**Conclusion:**

Our division of sperm-transfer behavior into sub-steps has provided a framework for the further detailed analysis of sperm transfer and its integration with other aspects of mating behavior. By determining the site of action of UNC-18 in sperm-transfer behavior, and its relation to the SPV sensory neurons, we have further defined the cells and tissues involved in the generation of this behavior. We have shown both a neuronal and non-neuronal requirement for UNC-18 in distinct sub-steps of sperm-transfer behavior. The definition of circuit components is a crucial first step toward understanding how genes specify the neural circuit and hence the behavior.

## Background

To execute a complex behavior, an animal must perceive and integrate information from various sources, process this information and perform the appropriate task. In many animals, one such complex behavior is mating, as different aspects of this behavior must be coordinated to ensure sexual reproduction. Male mating behavior in *C. elegans *affords the opportunity to study a complex multi-step behavior in an organism with a relatively simple nervous system.

When mating, *C. elegans *males exhibit a highly reproducible series of behavioral steps, culminating in the fertilization of hermaphrodite eggs [1-4]. Briefly, when the male comes in contact with a hermaphrodite, the male responds by placing the ventral side of his tail against the hermaphrodite, and moves backward along the body, scanning for the vulva. If the male's tail reaches the end of the hermaphrodite before encountering the vulva, the male turns and continues scanning the other side. When the vulva is located, the male ceases backward motion, prods with his copulatory spicules, inserts them into the vulva, and transfers sperm into the uterus [3-6]. Although the steps of reproductive behavior have been described in many organisms, relatively little is known about the genes that specifically control these behaviors [7].

We and others have begun molecular genetic analyses of the *C. elegans *male response [8-10], turning [4,11-13], vulval location [8-10,14], spicule insertion [12,15-17], and most recently, sperm-transfer [12] behaviors. By dissecting each step, their eventual integration as a complex series of overlapping sub-behaviors can be understood, and provide insight into nervous system function. In addition, the study of each individual step, each with its own unique aspects, may provide knowledge about the regulation of behavior not gotten from studying the other steps of mating. For example, the sperm-transfer sub-behavior in *C. elegans *is slower than any of the other mating steps, and does not occur instantaneously upon spicule insertion; therefore, its analysis will further our understanding of behavior.

To generate a behavior, an animal must employ its nervous system to perceive and integrate stimuli, and often coordinate this information with non-neuronal targets necessary for its proper execution. What is known about the neuronal contribution to sperm-transfer behavior comes from systematic ablation of the sexually dimorphic neurons in the male to assign them function during mating behavior [3]. Each of the two male spicules contains three neurons: two sensory neurons, designated SPV and SPD, and the motor neuron SPC [18]. Ablation of the SPV spicule neurons leads to premature sperm transfer at the vulva; therefore, the SPV neurons regulate the timing of sperm transfer by acting as a negative regulator [3]. Less is known about non-neuronal contribution to sperm-transfer behavior. Gower et al have recently shown that signaling mediated by inositol 1,4,5-triphosphate is required for efficient sperm transfer and propose that the site of action for this signaling is the male somatic gonad [12].

Here we present an analysis of the sperm-transfer step of male mating behavior. Direct observation suggests that sperm transfer comprises four sub-steps: initiation, release, continued transfer, and cessation. In a genetic screen for mutations affecting successful copulation, we isolated mutants in the sperm transfer process. Of the mutants we isolated, *unc-18(sy671*), the focus of this work, has defects in two distinct sub-steps of sperm transfer: initiation and continued transfer.

UNC-18 is a member of the SM family of highly conserved proteins involved in membrane-trafficking pathways, particularly in regulated exocytosis [19]. The mammalian *unc-18 *homologue (Munc-18) has been reported to be expressed in neurons, as well as in non-neuronal cell types [20-23]. In *C. elegans*, UNC-18 expression and function has been previously reported only in neuronal cells [24-26]. Here we show that, while the UNC-18 site of action for initiation involves neuronal expression, the continuation of transfer involves non-neuronal expression of UNC-18 in the male gonad.

## Results

### Steps of transfer and timing

**Figure 1:**
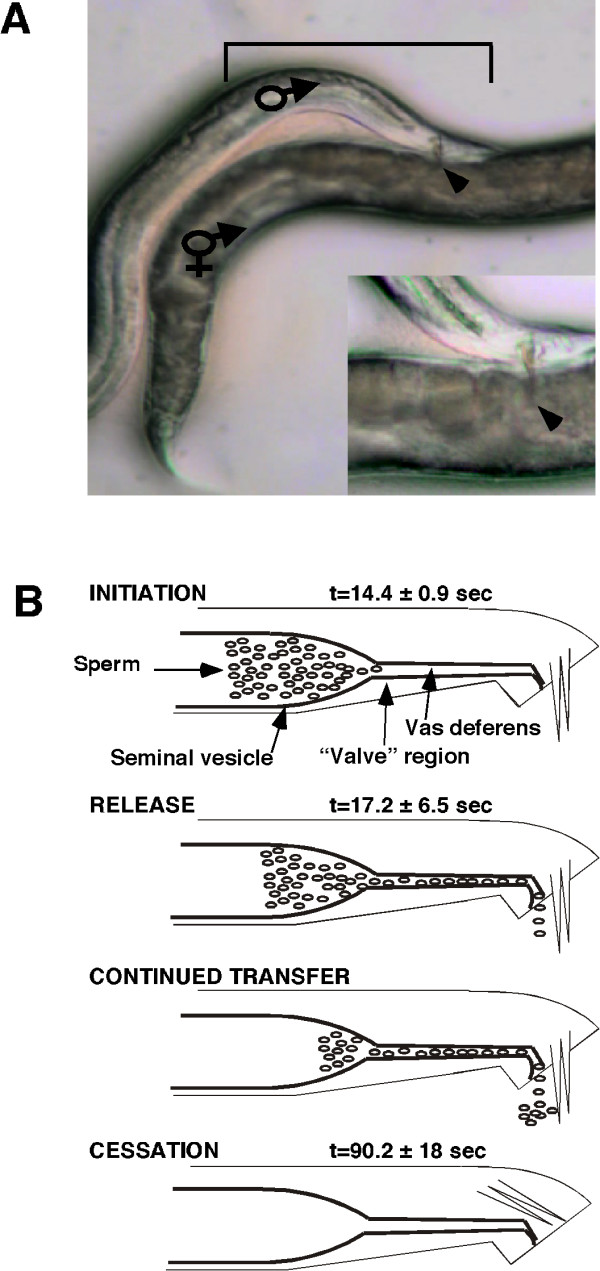
**Mating and the sub-steps of sperm transfer**. **(A) **A male with his tail positioned over a hermaphrodite's vulva after response, backing, turning, and vulva location. The inset shows the male tail after spicule insertion. Arrowhead denotes the tip of the spicules. The bracket denotes the approximate portion of the male shown in schematic in B. **(B) **Time (t) is equal to 0 when the spicules are tonically inserted into the vulva. The time it takes from t = 0 to completion of each sub-step is shown to the right of that step. Continued transfer was not timed, as it is simply the difference between the time from release to the cessation of transfer. For all times given, the minimum number of animals tested is 33. Note: 'valve' region is shown as open throughout this schematic as no morphological changes can be detected using the microscope we used to analyze sperm-transfer behavior.

As a first step toward understanding the molecular genetic processes underpinning sperm-transfer behavior and its regulation in *C. elegans*, and to establish a context to characterize sperm transfer mutants, we further defined this behavior. Our observation of wild-type sperm transfer and subsequent genetic screening (see below) has defined at least four steps in the transfer process: initiation, release, continued transfer, and cessation (Figure 1).

Once a male has inserted his spicules into the hermaphrodite's vulva, there is movement of sperm out of the seminal vesicle, which we call 'initiation'. The initiation step, from tonic insertion of spicules to sperm exit from the seminal vesicle, takes 14.4 ± 0.9 seconds (*n *= 33) (see Additional file 1). No gross changes in morphology were observed, except for the exit of sperm from the seminal vesicle, through the 'valve region', and into the tube formed by the vas deferens.

The sperm leave the seminal vesicle, travel through the vas deferens, and are released from the animal through the cloaca, a sub-step termed 'release' (see Additional file 2). The anal sphincter is contracted at this time, presumably to open the cloaca [18]. Release of sperm takes 17.2 ± 6.5 seconds (*n *= 34) from the time of insertion, and failure at this step causes sperm to collect in the vas deferens. Failure to release is rarely observed in wild-type animals; however, if a male's spicules fall out of the vulva while transferring sperm, sperm will sometimes collect in the vas deferens. Sperm continue to exit the seminal vesicle in a genetically separable step (see below) that we call 'continued transfer' (see Additional file 2). When all sperm that exit the seminal vesicle have been released, the spicules retract into the male. This is the 'cessation' sub-step and the termination of sperm transfer (see Additional file 3). The entire process from spicule insertion to cessation takes 90.2 ± 18 seconds (*n *= 33).

Liu and Sternberg reported that it took approximately 4 seconds, compared to our reported 90 seconds, to transfer sperm [3]. We attribute the discrepancy between the results to the microscope used for behavioral analysis (see Methods). Because the majority of sperm to be transferred are ejaculated in the beginning of the transfer process, it is difficult to observe without the use of a high-magnification dissecting microscope that as the behavior progresses, the male still continues to intermittently transfer a few sperm.

### Screen for mating behavior mutants

To begin to answer fundamental questions about how the sub-steps of sperm transfer are regulated, we performed a genetic screen designed to isolate males defective for this behavior. Briefly, *plg-1(e2001d) *males will extrude a gelatinous substance that puts a 'plug' over the hermaphrodite vulva after sperm transfer [27]. The *plg-1(e2001d) *allele is a dominant allele derived from a wild *C. elegans *isolate that was introgressed into the N2 wild-type strain. By screening for the lack of copulatory plugs in a clonal population of EMS mutagenized worms, we were able to isolate mutants defective in all steps of mating (see Methods). Secondary screening of putative mutants by observation of mating behavior allowed us to determine those mutants with defects in sperm transfer.

To isolate only those with non-developmental mating defects, we screened through the mutants we isolated for defects in morphology, focusing on those with wild-type anatomy. One recessive mutant isolated, *sy671*, is defective in the initiation step of sperm transfer. While *sy671 *mutant males will insert their spicules into the hermaphrodite vulva and remain tonically inserted for 82.9 ± 17 seconds (*n *= 14) (not statistically different by one-way analysis of variance (ANOVA) from wild type), they rarely initiate sperm transfer (Table 1). A second recessive mutant isolated, *sy672*, allows initiation and release, but is defective in the continuation of sperm transfer. *sy672 *males transfer very few sperm, although sperm are clearly visible in the seminal vesicle, and *sy672 *males keep their spicules tonically inserted during the transfer behavior for 62.6 ± 12 seconds (*n *= 15). Although the duration that *sy672 *males remain with their spicules inserted in the hermaphrodite vulva is less than wild type (significant using ANOVA), they should transfer significantly more sperm, as the bulk of transfer should have already occurred by this time. The *sy672 *mutant allows us to genetically separate the continued-transfer step from initiation and release steps. For the remainder of this study, we will focus on the analysis of *sy671*.

**Table 1:** *sy671 *phenotype and rescue

Genotype	Initiate/total^a^	Rescue^c^
Wild type^b^	40/40	N/A
*unc-18(sy671)*	1/39	N/A
*unc-18(sy671)*; *Ex[Punc18::unc-18::yfp]*	37/42	Yes
*unc-18(e81)*; *Ex[Punc18::unc-18::yfp]*	27/30	Yes
*unc-18(sy671)*; *Ex[Promoterless::unc-18::yfp]*	0/26	No
*unc-18(sy671)*; *Ex[Pint2itr-::unc-18::yfp (gonad)]*	0/84	No
*unc-18(sy671)*; *Ex[gpa-1::unc-18::yfp]*	3/93	No
*unc-18(sy671)*; *Ex[Punc119::unc-18::yfp (neuronal)]*	70/106	Yes
*unc-18(sy671)*; *Ex[myo-3::unc-18::yfp (muscle)]*	0/87	No
*unc-18(sy671)*; *Ex[aex-3::unc-18::yfp]*	29/34	Yes

^a ^For all genotypes carrying an extrachromosomal array (*Ex*), at least three independent lines were assayed except for *unc-18(sy671)*; *Ex[Promoterless::unc-18::yfp]*, for which only two lines each were tested. These results are the combined data from individual lines. See Additional file 4 for results of individual lines.

^b ^Wild type is strain PS3696, *plg-1(e2001)*; *him-5(e1490)*.

^c ^Rescue is defined as being different from *unc-18(sy671)*.

(*p *< 0.001) using ANOVA with a Tukey-Kramer multiple comparisons test (InStat3 software).

### *sy671 *acts downstream or in parallel to SPV

**Figure 2:**
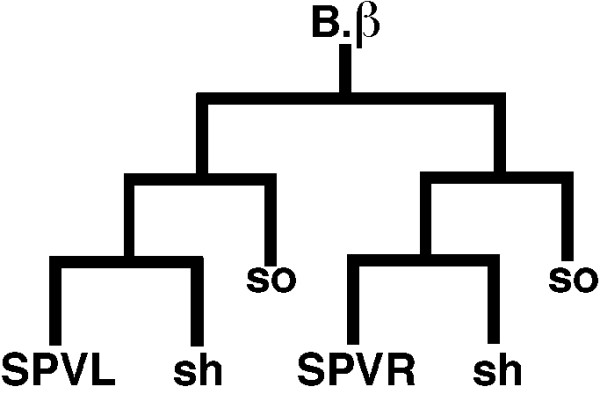
**The B.β lineage**. The B.β lineage gives rise to both SPV neurons, left (L) and right (R), as well as two sheath (sh) cells and two socket (so) cells.

As mentioned above, the SPV neurons regulate sperm transfer by inhibiting release until the spicules have penetrated the vulva: SPV-ablated wild-type males prematurely transfer sperm before spicule insertion [3]. Because *sy671 *has the opposite phenotype (failure to initiate sperm transfer after spicule insertion) we ablated the SPV neurons in the *sy671 *mutant to see if this could suppress the initiation defect. In particular, we ablated the B.β cell, the precursor of both SPV neurons (SPVL, SPVR) (Figure 2). Although the B.β ablation also eliminates four of 12 structural cells (two sheath and two socket), the sheath cells are not necessary for spicule morphology, and the function of the socket cells is redundant [28]. In addition, ablation of the B.β cell causes the same defect in sperm transfer as the ablation of the SPV alone [3]. We found that ablation of the B.β cell (SPV neurons) in the *sy671 *mutant did not suppress the initiation defect (Table 2). This observation suggests that the defect in our mutant lies downstream or in parallel to the SPV regulation in the sperm-transfer pathway.

**Table 2:** Results of the ablation of B.β.

Relevant Genotype	Cell Ablated	n^a^	Premature Transfer^b^	Wild-type Transfer	No Transfer	Percent Altered^c^
Wild-type^d^	none	10	0	10	0	-
Wild-type	B.β	14	5	8	1	42.9
*Sy671*	none	11	0	0	11	-
*Sy671*	B.β	9	0	0	9	0

^a ^Number of animals assayed.

^b ^Scored as animals that transfer sperm without tonic contraction of spicules in the vulva.

^c ^Percentage altered is the number of animals with altered transfer divided by the total number of animals tested. Altered transfer for wild type includes premature transfer and no transfer. Altered transfer for *sy671 *includes premature transfer and wild-type transfer.

^d ^Wild type is strain PS3696, *plg-1(e2001)*; *him-5(e1490)*.

### Molecular characterization of *sy671*

**Figure 3:**
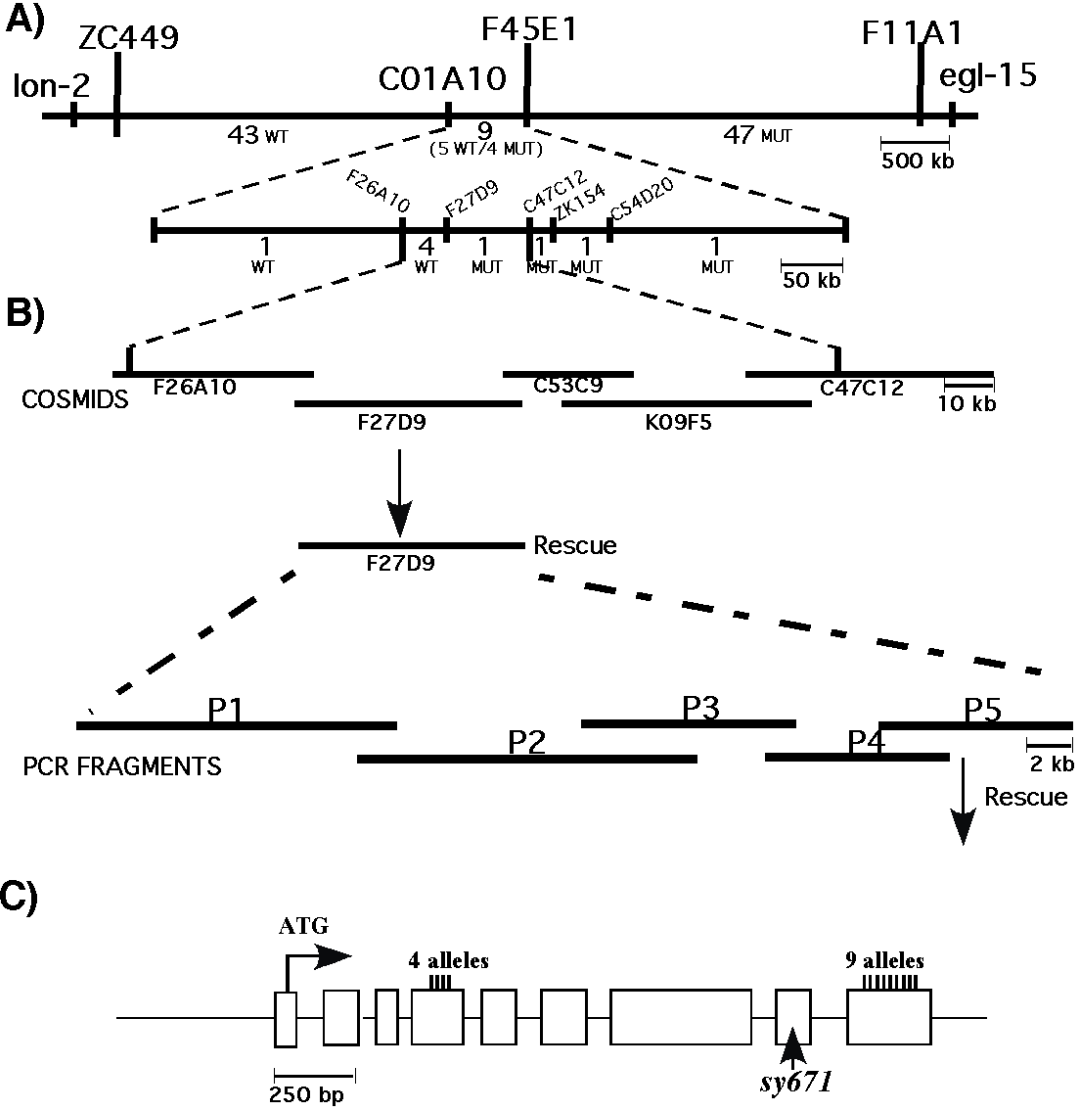
**Mapping and cloning of *unc-18(sy671)***. **(A, B) **Summary of the cloning of *sy671*. **(A) **Initial SNP mapping placed *sy671 *on chromosome X between markers ZC449 and F11A1. Three-factor mapping using *lon-2 *and *egl-15 *flanking *sy671 *allowed for isolation of 99 Lon non-Egl recombinants. Numbers between the markers represent the number of recombinants isolated between those markers and the phenotype of those worms when made homozygous. **(B) **The cosmid clones covering the interval containing *sy671 *that were transformed in pair-wise combination are shown. F27D9 transformed alone was able to rescue *sy671*. The five PCR products used to further define the locus are shown, with P5 being able to rescue when transformed into *sy671 *mutants. **(C) **The genomic organization of *unc-18 *(boxes represent exons). Position of the putative start ATG is shown and the genomic region from start to stop is 2.3 kb. The position of the molecular lesions in 13 previously identified alleles, and the change in *sy671*, are indicated. The numbering is based on *C. elegans *isoform A (WormBase WS130).

To understand the sperm-transfer initiation defect in *sy671 *males, we cloned this locus. We employed single-nucleotide polymorphism (SNP) mapping [29] to map *sy671 *to a 120-kb interval between the polymorphisms F26A10:1544 and C47C12:6306 (see Methods; Figure 3A and 3B). Injection of cosmids and PCR fragments narrowed the *sy671 *locus to a 7.2-kb region containing F27D9.1 (previously identified as the *unc-18 *locus). We sequenced the predicted coding and non-coding regions of *unc-18 *from the *sy671 *mutant, and found that the *sy671 *mutant contains a G→A missense mutation in an *unc-18 *exon, which changes the amino-acid Arg476 to His (see Methods).

In *C. elegans*, *unc-18*, which encodes a member of the Sec1/Munc-18 family of proteins, is required in neurons for synaptic-vesicle exocytosis [30,31]. For exocytosis to occur, secretory vesicles must dock, be primed, and then fuse with the plasma membrane [32-34]. UNC-18 has been proposed to function in each of these steps of exocytosis, based on work in various systems, and may also act as a trafficking factor for other proteins necessary for exocytosis [21,35-38]. Although UNC-18 has been postulated to act in various steps of the exocytosis pathway, in *C. elegans *it has been shown that there is a reduction of docked vesicles at the active zone in *unc-18 *mutants, indicating that UNC-18 functions at least as a facilitator of vesicle docking in *C. elegans *[39].

Mutations in *unc-18 *cause locomotor defects and acetylcholine accumulation, and therefore UNC-18 has been implicated in the release of acetylcholine [24,25,40]. UNC-18 interacts with syntaxin, a protein believed to mediate fusion of synaptic vesicles to the plasma membrane and hence neurotransmitter release [26,41]. While most alleles of *unc-18 *have severe locomotor defects, *sy671 *males have only mild locomotor defects (data not shown). Although the amino-acid change in *sy671 *(Arg476 to His) occurs in a residue conserved among UNC-18 orthologs (data not shown), this residue lies outside the syntaxin-binding region [37].

We were intrigued by the unique position of the molecular lesion in the *sy671 *allele of *unc-18*. All reported sequenced alleles of *unc-18 *have a molecular defect in either exon 4 or exon 9 [31]; however, *sy671 *has its defect in exon 8 (Figure 3C). Of the other *unc-18 *mutants, only two, *md1401 *and *md1264*, move sufficiently well to allow sperm transfer to be assayed. Although their movement is indistinguishable from *sy671*, both mutants are able to initiate transfer (data not shown). This difference among alleles leads us to speculate that the mutation in *sy671 *has some specificity for sperm transfer through a mechanism different from the function of UNC-18 in vesicle release.

To address if a perturbation of vesicle release is the likely defect in *sy671 *males, we assayed males that were transheterozygous for *sy671 *and for a severe allele of *unc-18*, *b403*. The *unc-18(b403) *allele has a single amino-acid substitution (exon 9) that causes the accumulation of acetylcholine, the inability to bind syntaxin, and a severe locomotor defect, all characteristics of defective vesicle release [31]. If *sy671 *does have a defect in a yet uncharacterized UNC-18 function, we would expect that when placed in *trans *to an allele that perturbs vesicle release through a single amino-acid substitution, we would see compensation for the sperm-transfer defect. However, the *b403 *allele fails to complement *sy671 *for the initiation of transfer (0/16 animals initiated sperm transfer), and therefore it seems likely that the mutation in *sy671 *also alters vesicle release.

### *unc-18 *is expressed in the male gonad and nervous system

**Figure 4:**
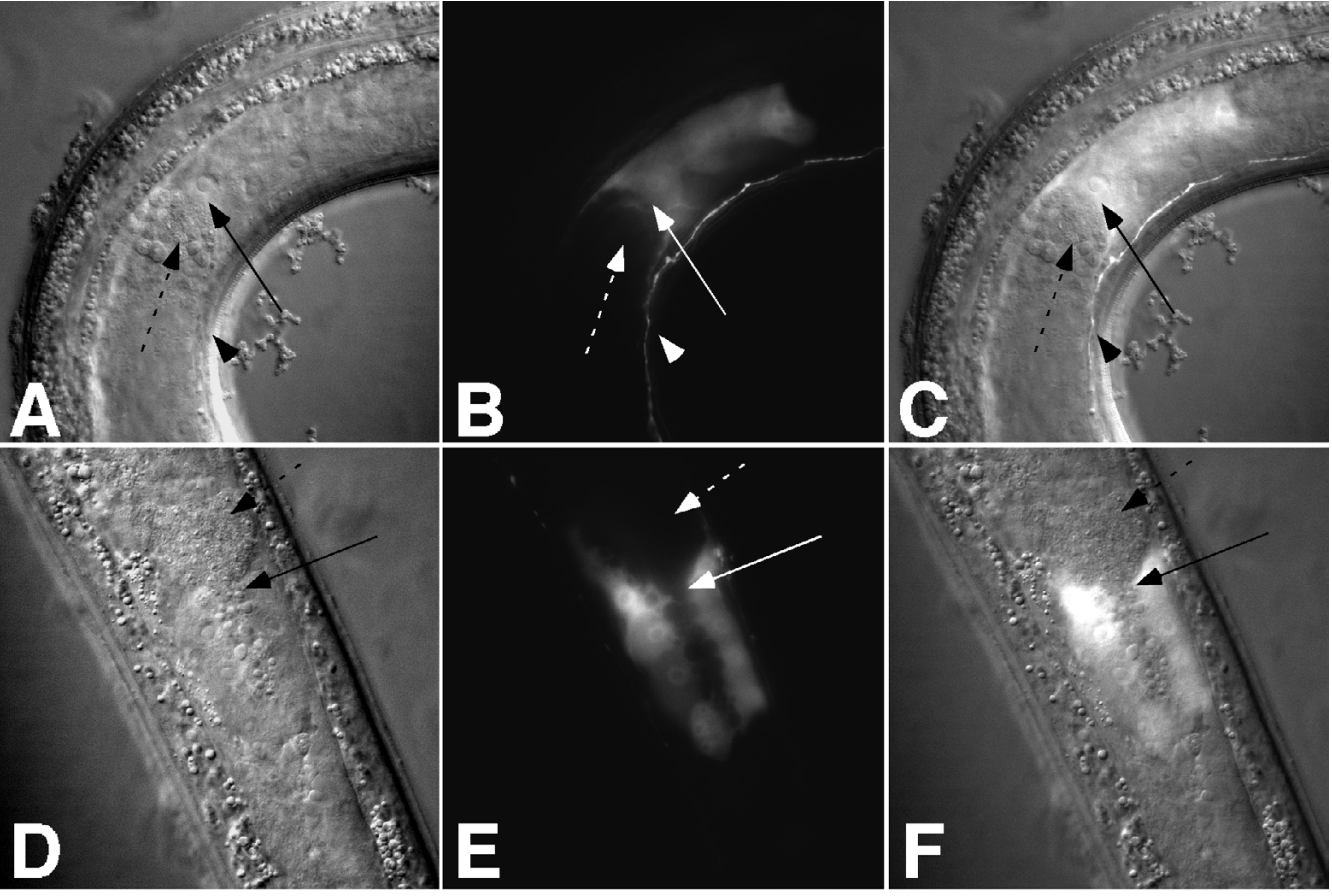
***unc-18 *expression in the male gonad and ventral nerve cord**. Nomarski **(A, D)**, fluorescence **(B, E) **and merged **(C, F) **images of *unc-18::YFP *expression in the male gonad region and part of the ventral nerve cord. **(A–C) **Lateral view; **(D–F) **dorsal view. The dashed arrow indicates the sperm in the seminal vesicle, and the solid arrow indicates the valve area where the sperm exit into the vas deferens. Arrowheads in **(A–C) **indicate the ventral nerve cord.

**Figure 5:**
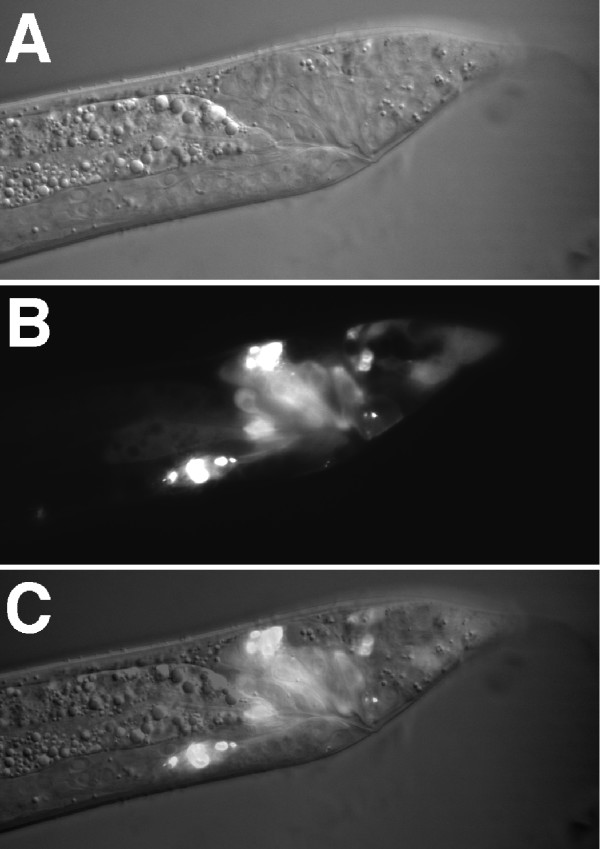
***unc-18 *expression in the developing male tail**. Nomarski **(A)**, fluorescence **(B) **and merged **(C) **images of *unc-18::YFP *expression in the developing male tail (L4). Animals are *pha-1(e2123ts) *young adult males transformed with the extrachromosomal array *pha-1*+*unc-18::YFP*.

Previous characterization of UNC-18 expression using polyclonal antisera showed that UNC-18 was present in ventral-cord motor neurons and some unidentified head neurons in the adult hermaphrodite [25]. To replicate the expression in the hermaphrodite and determine expression in the male, we fused the *unc-18 *upstream sequences used in the mutant-rescue experiment to a YFP reporter. In transgenic animals, this reporter (*unc-18::YFP*) recapitulated the previously observed expression in the hermaphrodite. In the male, *unc-18::YFP *showed the same expression in the ventral cord and head, but surprisingly also had strong expression in the gonad. The expression was strongest in the 'valve region', a non-neuronal tissue that lies at the junction of the seminal vesicle and the vas deferens (Figure 4). Expression was also present in most, if not all neurons in the male tail (Figure 5).

### Site of action: initiation

To define the site of action of *unc-18 *in sperm-transfer behavior, we created a YFP-tagged version of UNC-18 under the regulation of the *unc-18 *upstream regulatory region (*unc-18::UNC-18::YFP*), which was able to rescue the *sy671 *sperm transfer initiation defect (Table 1). This construct was also able to confer the null allele, *unc-18(e81)*, with the ability to initiate sperm transfer (Table 1). Next we expressed the *UNC-18::YFP *functional protein under different tissue-specific, transcriptional-control regions to define its site of action in sperm-transfer behavior, beginning with the two most probable candidates: the gonad and the spicule neurons.

Because *unc-18 *expression in the male gonad coincides with the region where sperm exit from the seminal vesicle to the vas deferens, this seemed a likely candidate for the site of action of *unc-18 *in the initiation of sperm-transfer behavior. An equally attractive candidate is the SPV spicule neurons in the male tail, as they are known regulators of sperm transfer.

**Figure 6:**
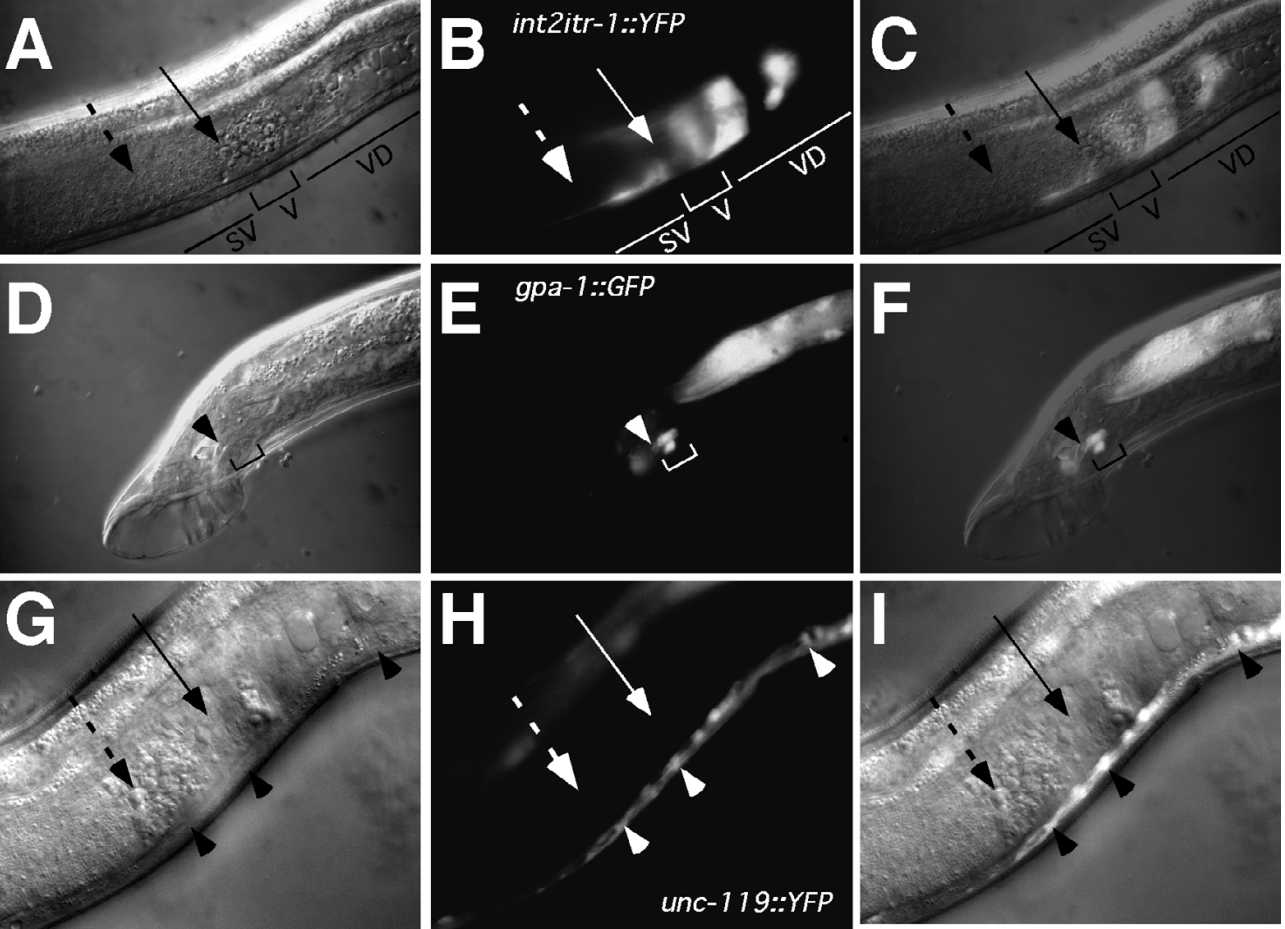
**Expression of reporter constructs in males**. **(A–C) **The *int2itr-1 *regulatory region drives expression in the gonad of males. Nomarski **(A) **and fluorescence **(B) **images of the male gonad region that expresses the *int2itr-1::YFP *reporter construct (lateral view, anterior left). An overlap of these images is shown in **(C)**. **(A–C) **are *pha-1(e2123ts) *young adult males transformed with the extrachromosomal array *pha-1*+*int2itr-1::YFP*. The dashed arrow indicates the sperm in the seminal vesicle (SV) and the solid arrow indicates the valve area (V) where the sperm exit into the vas deferens (VD). **(D–F) **The *gpa-1 *regulatory region drives expression in spicule neurons. Nomarski **(D) **and fluorescence **(E) **images (dorsolateral) of the male tail showing expression of the *gpa-1::GFP *construct in the two neurons (SPV and SPD, bracket) that innervate the spicules. The top of the spicule is denoted by an arrowhead, with autofluorescence of the spicules posterior to the arrowhead. An overlap of these images is shown in **(F)**. Animal shown is a *pha-1(e2123ts) *young adult male transformed with the extrachromosomal array *pha-1*+*gpa-1::GFP*. (G–I) The *unc-119 *regulatory region drives expression in ventral cord neurons, but not in the gonad. Nomarski **(G) **and fluorescence **(H) **images of the male gonad region and part of the ventral nerve cord that expresses the *unc-119::YFP *reporter construct (lateral view, anterior left). An overlap of these images is shown in **(I)**. No expression is seen in the gonad. The dashed arrow indicates the sperm in the seminal vesicle, and the solid arrow indicates the area where the sperm exit into the vas deferens. Arrowheads indicate neurons in the nerve cord. Animal shown is a *pha-1(e2123ts) *young adult male transformed with the extrachromosomal array *pha-1*+*unc-119::YFP*.

To express *unc-18 *under a gonad-specific promoter, we used a portion of the described *itr-1 *gene [42]. It was previously reported that two introns and one exon of the *itr-1 *gene (including a 2.1-kb fragment that includes the C-terminal six codons, the 3' untranslated region, and downstream sequences) gave expression in the intestine, pharyngeal isthmus, and gonad of hermaphrodites [42]. Through further dissection of this region, called the pC promoter, we were able to drive expression (using only intron 2) in the seminal vesicle, the valve region, and the vas deferens of the male (Figure 6A–C). This expression coincides with that described for the entire pC promoter in the male [12]. We call this element *int2itr-1*. We then expressed *unc-18 *under the control of the *itr-1 *gonadal enhancer (*int2itr-1::UNC-18::YFP*), but were not able to rescue the mutant phenotype in *sy671 *(Table 1). To express *unc-18 *under a spicule neuron promoter, we used 3 kb of 5' upstream sequence of the *gpa-1 *gene that drives expression in all three types of spicule sensory neurons, including the SPV neurons [28] (Jiang LI, Mendel JE and Sternberg PW, unpublished observations; Figure 6D–F). The *gpa-1::UNC-18::YFP *transgene was also unable to rescue the sperm-transfer initiation defect in *sy671 *(Table 1).

After initial testing of possible gonadal and spicule neuron contributions, we chose to express *unc-18 *more broadly to see if *unc-18 *was at least acting in neurons to initiate the transfer of sperm. We used a pan-neuronal promoter (*unc-119*) [43] to drive expression of *unc-18 *(*unc-119::UNC-18::YFP*) in neurons (Figure 6G–I), and also used a body-wall muscle promoter (*myo-3::UNC-18::YFP*) to rule out non-specific rescue effects [44]. The neuronal expression of *unc-18 *was able to rescue the initiation defect (Table 1). These results suggest that although *unc-18 *is expressed in the male gonad, its expression in this tissue is not required for the initiation of sperm transfer, but it is required in neurons. To confirm this result, we used the 5' regulatory region of the *aex-3 *gene, another pan-neuronal promoter, to express *unc-18 *(*aex-3::UNC-18::YFP*) [45]. As expected, this transgene was able to rescue the sperm-transfer initiation defect in *sy671 *(Table 1). The negative result using *gpa-1::UNC-18::YFP *to drive expression in the spicule neurons does not exclude an *unc-18 *contribution from these neurons; therefore, we decided to further define the site of action of UNC-18 in this behavior using various neuronal promoters.

UNC-18 was initially described to be expressed in all ventral-cord motor neurons [25]. The ventral cord has been divided into eight classes of motor neurons: four classes that innervate ventral muscles (VA, VB, VC, VD) and four classes that innervate dorsal muscles (DA, DB, DD, AS) [46-49]. The D-type ventral cord neurons (VD and DD) are gamma-aminobutyric acid (GABA)ergic and the others (VA, VB, VC, DA, DB, AS) are cholinergic [49,50]. The VC cholinergic neurons are hermaphrodite-specific, while in males, the homologous cells divide to give the male-specific CP and CA motor neurons [18,47].

To test whether *unc-18 *was acting exclusively in either cholinergic or GABAergic ventral cord motor neurons to initiate sperm transfer, we drove expression specifically in each type. To express *unc-18 *in all GABAergic neurons in the *sy671 *mutant, we used the promoter region from the *unc-25 *gene to drive expression (*unc-25::UNC-18::YFP*) [51]. Expression in only the GABAergic neurons was unable to rescue our mutant (Table 3).

**Table 3:** Neuronal promoters

Genotype	Initiate/total^a^	Rescue^b^
*unc-18(sy671)*; *Ex[unc-25::unc-18::yfp]*	0/83	No
*unc-18(sy671)*; *Ex[unc-17::unc-18::yfp]*	ND^c^	
*unc-18(sy671)*; *Ex[cho-1::unc-18::yfp]*	ND^c^	
*unc-18(sy671)*; *Ex[acr-2::unc-18::yfp]*	27/98	Yes
*unc-18(sy671)*; *Ex[unc-4::unc-18::yfp]*	2/86	No
*unc-18(sy671)*; *Ex[acr-5::unc-18::yfp]*	49/66	Yes

^a ^For all genotypes tested, at least three independent lines were assayed. These results are the combined data from individual lines. See Additional file 5 for results of individual lines.

^b ^Rescue is defined as being different from *unc-18(sy671) *in Table 1.

(*p *< 0.001) using ANOVA with a Tukey-Kramer multiple comparisons test (InStat3 software).

^c ^ND, not done. These lines were not assayed because the introduction of the transgene caused severe mating behavioral defects in steps prior to sperm transfer assay.

To express *unc-18 *in all cholinergic neurons in the *sy671 *mutant, we initially tried using promoters from both *unc-17 *[52] and *cho-1*[53] to drive expression. However, the use of either of these promoters (*unc-17::UNC-18::YFP *or *cho-1::UNC-18::YFP*), caused penetrant locomotion and mating behavioral defects in the steps prior to sperm transfer, and therefore the worms could not be assayed for their ability to transfer sperm. These defects, not seen in non-transgenic worms, could be caused by overexpression of UNC-18 or possible titration of promoter regulatory proteins caused by our transgenic arrays.

Next we tried the *acr-2 *promoter, which drives expression in the VA, VB, DA, and DB cholinergic ventral-cord motor neurons [54]. The *acr-2 *construct, *acr-2::UNC-18::YFP*, when expressed in *sy671 *mutants, was able to rescue initiation (Table 3). Because *acr-2 *is expressed in cholinergic neurons throughout the ventral cord and not in the male spicule sensory neurons (data not shown), these ventral-cord motor neurons are strong candidates for contributing to the initiation of sperm transfer.

To further test ventral-cord input for the initiation of sperm-transfer behavior and to further pinpoint the site of action to a specific subset(s) of cells, we used additional promoters to attempt to rescue the *sy671 *defect. We initially tested two classes of cholinergic ventral-cord motor neurons, the A-type neurons, VA and DA (necessary for backward movement) and the B-type neurons, VB and DB (necessary for forward movement) [55]. We used the *unc-4 *upstream sequences to drive expression in the A-type neurons [56] and the *acr-5 *promoter to drive expression in B-type neurons [57]. By expressing *unc-18 *under the *acr-5 *regulatory region, we were able to rescue the *sy671 *initiation defect (Table 3), but were unable to do so with the *unc-4 *promoter (Table 3). This result suggests that *unc-18 *in the B-type neurons (VB and/or DB) could be necessary for initiation, again with the exception of the neurons outside the ventral cord that express *acr-5*.

**Figure 7:**
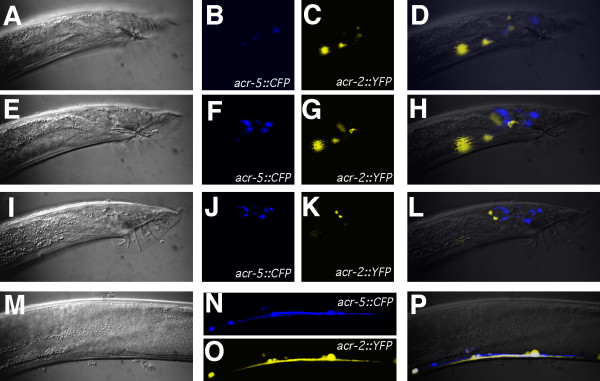
***acr-2::YFP *and *acr-5::CFP *expression overlaps in the ventral cord, but not in the tail of the male**. Lateral views with anterior oriented left, and dorsal oriented upwards **(A–L) **expression of *acr-5::CFP *and *acr-2::YFP *in the male tail at three focal planes: **(A–D)**, **(E–H) **and **(I–L)**. Nomarski **(A, E, I)**, *acr-5::CFP *fluorescence **(B, F, J)**, *acr-2::YFP *fluorescence **(C, G, K) **and merged **(D, H, L) **images. **(M–P) **overlap of *acr-2::YFP *and *acr-5::CFP *in the ventral cord. Nomarski **(M)**, *acr-5::CFP *fluorescence **(N)**, *acr-2::YFP *fluorescence **(O) **and merged **(P) **images. Animals shown are *pha-1(e2123ts) *young adult males transformed with the extrachromosomal array *pha-1*+ *acr-2::YFP *and carrying the extrachromosomal array *acr-5::CFP *that was crossed into the strain.

We determined that *acr-5 *is expressed in the male-specific ray sensilla (rays 2, 3, 4, 6, and 9) using the *acr-5 *regulatory region to drive expression of *DsRed2 *(*acr-5::DsRed2*). The expression in the rays appears to be in the A-type ray neurons, as *acr-5 *expression in the tail does not overlap with *pkd-2::GFP*, which is expressed in the B-type ray neurons (data not shown) [10]. *acr-5 *also shows expression in a cell in the right preanal ganglion, which we have tentatively identified as PGA or PVZ. However, input from the *acr-5 *expressing cells in the male tail in initiating transfer is very unlikely, as there is no overlap with *acr-2 *expression in the tail (Figure 7A–L), yet both promoters driving *unc-18 *expression can rescue the initiation defect in *unc-18(sy671) *mutants. The overlap in expression of *acr-2 *and *acr-5 *is in the ventral cord (Figure 7M–P), making a strong case for it being necessary for the initiation of sperm transfer. Although these data also suggest it is specifically the B-type motor neurons in the ventral cord that are relevant for the initiation of sperm transfer, further experiments indicate that the male-specific CA ventral-cord motor neurons are stronger candidates (see below).

**Figure 8:**
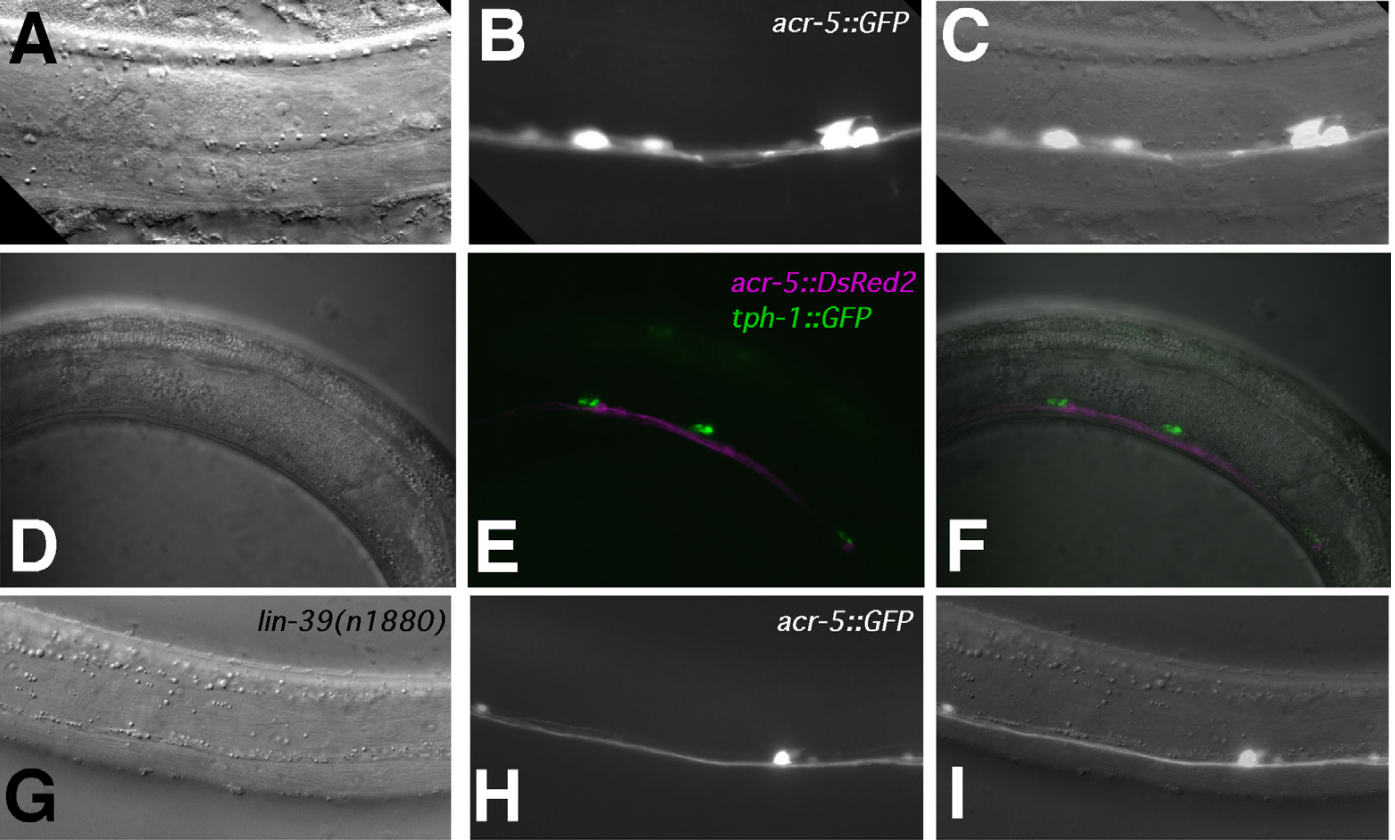
***acr-5 *promoter-driven expression in the male ventral cord motor neurons**. **(A–C) ***acr-5::GFP *expression in wild-type males in the ventral cord of the posterior mid-body region. Ventral view with anterior oriented left. Nomarski **(A)**, fluorescence **(B) **and merged **(C) **images. Animal shown is a *pha-1(e2123ts) *young adult male transformed with the extrachromosomal array *pha-1*+ *acr-5::GFP*. **(D–F) ***tph-1::GFP *(green) and *acr-5::DsRed2 *(red) expression in the male ventral cord. Nomarski **(D)**, merged *tph-1::GFP *and *acr-5::DsRed2 *fluorescence **(E)**, and merged **(F) **fluorescence and Nomarski images. Lateral views with posterior oriented left, and dorsal oriented upwards. Animal shown is a *pha-1(e2123ts) *young adult male transformed with the extrachromosomal array *pha-1*+ *acr-5::DsRed2 *and carrying the extrachromosomal array *tph-1::GFP *that was crossed into the strain. **(G–I) ***acr-5::GFP *in *lin-39 (n1880) *mutant males in the ventral cord of the posterior mid-body region. Ventral view with anterior oriented left. Nomarski **(G)**, fluorescence **(H)**, and merged **(I) **images. Animal shown is a young *lin-39(n1880) *mutant adult male that carries the same transgene used in **(A–C) **because it was crossed into the strain.

In the adult male ventral cord we noticed that *acr-5::YFP *was expressed not only in the B-type neurons, but also in additional neurons (Figure 8A–C). Because the male has additional ventral cord neurons, the CPs and CAs, we thought that these may account for the additional cells showing expression, and it may be these cells that are necessary for initiation of sperm transfer. Because the CP neurons are necessary for efficient turning behavior [4], we wanted to test if the *acr-5*-expressing cells were CP neurons. We used a strain carrying a *tph-1::GFP *transgene that drives expression in the CP neurons [58], and crossed it into animals expressing *acr-5::DsRed2*. The additional cells in the male ventral cord that express *acr-5::DsRed2 *did not overlap with the CP-expressing cells, but in many cases these cells were anterior to the CP neurons, suggesting that they could be CA neurons [18] (Figure 8D–F). Because there are no reported markers for the CA neurons, we tested the possible contribution of these neurons to sperm-transfer initiation by assaying *lin-39 *mutant males.

The *lin-39 *gene, which encodes a homeobox gene, is required for mid-body region-specific development in *C. elegans *[59,60]. In wild-type males, P(3–11).aap divide during late L3 to generate male-specific motor neurons. The posterior cells, P(3–11).aapp, are the CP neurons (CP1–9) and the anterior cells, P(3–11).aapa, are the CA neurons (CA1–9) [18]. In *lin-39 *mutant males, P(3–6).aap (the precursors for CA1–4 and CP1–4) do not divide, but instead have a compact neuron-like morphology or die [60]. Also affected in *lin-39 *mutant males are P(7–8).aapp (CP 5, 6), which take on a more posterior fate, as judged by the loss of serotonin staining in CP5 and CP6 [60]. This observation suggests that P(7, 8).a might be transformed into P9.a-like cells [60], thus also affecting CA5 and CA6.

If the CA neurons (1–4 and possibly 5, 6) are necessary for initiation of sperm transfer, we would expect *lin-39 *males to be unable to initiate this behavior, as these cells have been genetically ablated or misfated. We tested two alleles of *lin-39 (n1760 *and *n1880*, the putative null) and both were unable to initiate sperm transfer (Table 4), further supporting a role for the CA neurons and confirming ventral cord involvement, limiting it to the mid-body region. To further corroborate that the unassigned ventral cord motor neurons expressing *acr-5 *in the wild-type males could be CA neurons, we analyzed the expression of *acr-5::GFP *in *lin-39 *mutant males. As expected, we saw fewer cells expressing *acr-5::GFP *in the ventral cord of *lin-39 *mutant males (Figure 8G–I).

**Table 4:** lin-39 males do not initiate

Genotype	Initiate/total
*him-5 (e1490)*	16/16
*unc-18(sy671)*	1/39
*lin-39 (n1880)*; *him-5 (e1490)*	0/16
*lin-39 (n1760)*; *him-5 (e1490)*	0/8

### Site of action: continued transfer

Although the *sy671 *mutants can be rescued for the initiation of sperm transfer using a transgene to drive neuronal expression of UNC-18 in a subset of ventral cord cholinergic neurons (most likely the CA neurons), these animals did not transfer as many sperm as wild type. We observed that by using the *unc-119 *promoter to drive *unc-18 *expression, the animals would initiate, and usually the sperm that were released from the seminal vesicle were released from the animal, but the animals transferred much less sperm and thus appeared to be defective in the continuation of transfer. This observation raised the possibility that UNC-18 functions in two separate sub-steps of the sperm-transfer process (both the initiation and the continuation of transfer), and that neuronal expression of *unc-18 *was responsible for the initiation of transfer, while the gonad expression may be necessary for continued transfer.

To formally test this possibility, we hypothesized that if we could add back both neuronal and gonadal expression of *unc-18*, using different 5' control regions, we should restore wild-type sperm transfer to the *unc-18(sy671) *mutants. To do this, we again used our neuronal (*unc-119::UNC-18::YFP*) construct, and remade our gonadal transgene with a CFP marker (*int2itr-1::UNC-18::CFP*) to facilitate the scoring of worms carrying both transgenes. To quantify the amount of sperm transferred, we devised a 'continuation assay' (Methods). This assay uses the percentage of cross progeny as a proxy for the amount of sperm transferred.

As a control for the effectiveness of our continuation assay, we tested *unc-18*(*sy671*) mutants carrying a rescuing transgene (*unc-18::UNC-18::YFP*) to determine if they could sire a wild-type percentage of cross progeny. Not only did we observe that these transgenic animals appeared to transfer a similar amount of sperm to that of wild type, but as expected they sired an equivalent percentage of cross progeny (see Additional file 6). This construct also was able to completely restore wild-type sperm transfer in the *unc-18 *null mutant (see Additional file 6). In addition, as expected based on observation of sperm transfer, animals carrying the *unc-119*-driven transgene sired much fewer progeny than wild type in our assay (see Additional file 6), because they initiated transfer and released sperm, but did not continue to transfer. In addition, lines carrying only gonadally expressed UNC-18 (*int2itr-1::UNC-18::CFP*) did not sire cross progeny (data not shown), consistent with an inability to initiate transfer.

We then tested if we could restore wild-type sperm transfer by expressing *unc-18 *in both neuronal (*unc-119::UNC-18::YFP*) and gonadal (*int2itr-1::UNC-18::CFP*) tissues. Indeed, when *unc-119 *and *int2itr-1 *promoter elements are used to drive *unc-18 *expression simultaneously in the *sy671 *mutant, sperm transfer is restored completely (see Additional file 6). These transgenes are lost at some frequency during meiotic cell divisions [61], and if either transgene is lost, wild-type sperm transfer no longer occurs in that animal (see Additional file 6): if the *unc-119::UNC-18::YFP *transgene is lost, animals do not initiate, and if the *int2itr-1::UNC-18::CFP *transgene is lost animals will initiate, but no longer continue to transfer and hence sire fewer crossprogeny. The results from the continuation assays support our hypothesis that UNC-18 functions in two different tissues to mediate two sub-steps of sperm transfer.

**Figure 9:**
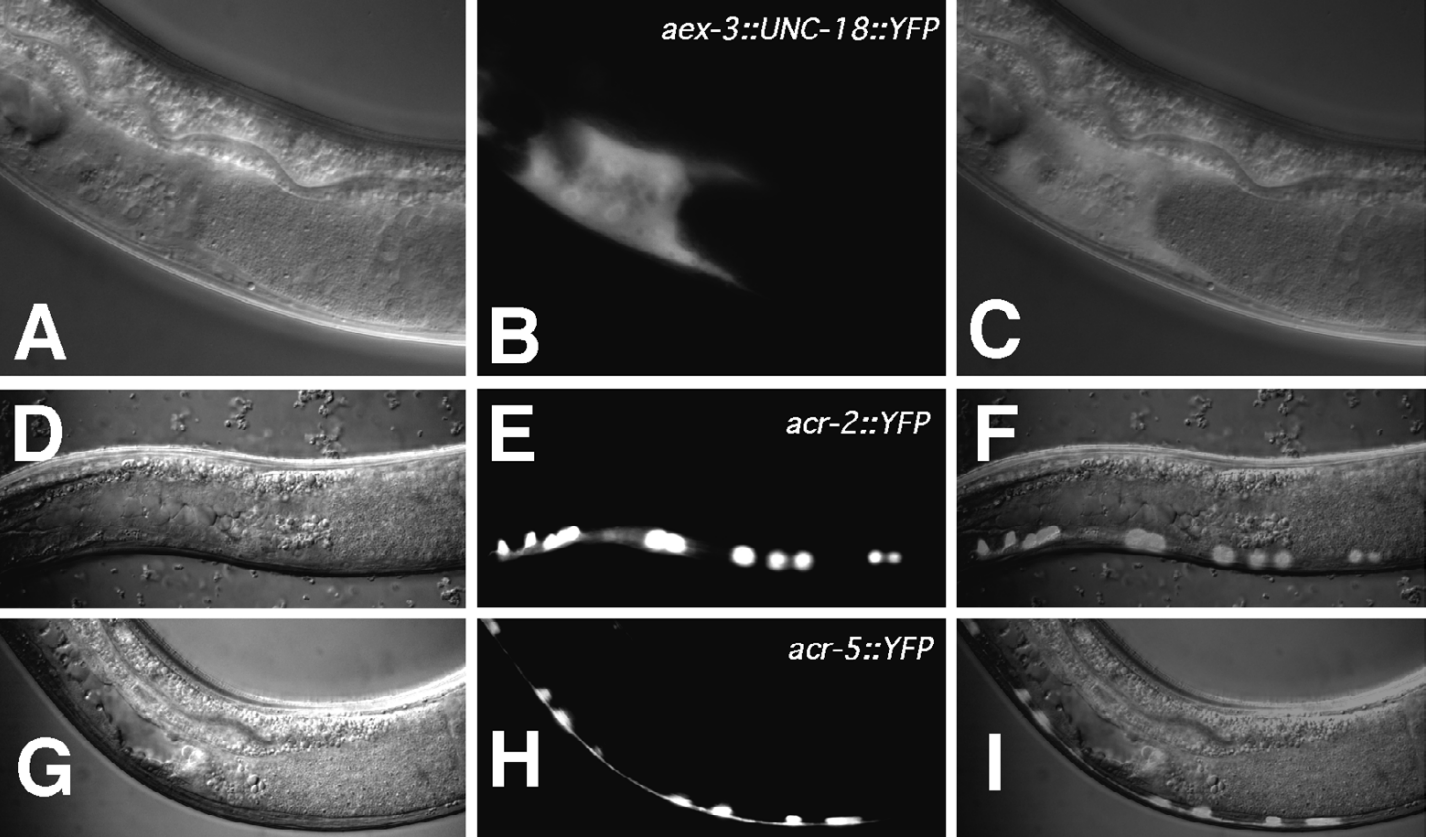
**Expression of reporter constructs in the male**. Lateral views and all images are oriented such that posterior is left and dorsal is up. Images in **(A–C) **were inverted to obtain this orientation. **(A–C) ***aex-3::UNC-18::YFP *expression in the male. Nomarski **(A)**, fluorescence **(B)**, and merged **(C) **images. Slightly higher magnification than images in **(D–I)**. Although *aex-3 unc-18::YFP *is expressed in the ventral cord, in **(A–C) **we focused on the plane that best showed the gonad expression. **(D–F) ***acr-2::YFP *expression in the male. Nomarski **(D)**, fluorescence **(E) **and merged **(F) **images. **(G–I) ***acr-5::YFP *expression in the male. Nomarski **(G)**, fluorescence **(H)**, and merged **(I) **images. Animals shown are *pha-1(e2123ts) *young adult males transformed with the extrachromosomal array *pha-1*+ *reporter::YFP*.

We also assayed *unc-18(sy671) *animals carrying either the *acr-2::UNC-18::YFP*, the *acr-5::UNC-18::YFP*, or the *aex-3::UNC-18::YFP *transgene using our continuation assay. Both *acr-2 *and *acr-5*-driven transgenes sired fewer progeny as expected, similar to our results using the *unc-119 *regulatory region, however *aex-3::UNC-18::YFP *was able to fully restore wild-type transfer. We then checked to see if *aex-3:: unc-18::YFP *was also expressed in the male gonad, which would explain the complete rescue. *aex-3:: unc-18::YFP *is indeed expressed in the gonad, while *acr-2::YFP *and *acr-5::YFP *are not (Figure 9 and data not shown). Although *sy671 *mutant animals carrying the *acr-5::UNC-18::YFP *transgene mostly transferred less sperm, occasionally an animal transferred wild-type amounts. Considering that the *acr-5 *regulatory region we used does not drive detectable expression in the gonad, expression in the gonad may not be absolutely required, but important for proper continued transfer.

One caveat for the use of the continuation assay as a proxy for sperm transfer is that the sperm being transferred must be fertile. Mature spermatozoa that are capable of fertilization develop from spermatids following ejaculation by the male [62]. As it is possible to activate spermatids *in vitro *to test whether they are competent to mature (through the addition of exogenous proteases [62]), we isolated and activated sperm from *unc-18(sy671) *males. Spermatids from *unc-18(sy671) *males were capable of developing into mature spermatozoa and there appeared to be similar quantities of spermatids released upon dissection compared with wild-type males (data not shown). This observation suggests that there is no sperm formation or activation defect in this mutant.

Although the sperm from *unc-18(sy671) *males are capable of being activated *in vitro*, we cannot rule out the possibility that the vas deferens in these mutant males does not release the secreted material necessary to activate the sperm *in vivo*. Because UNC-18 is involved in regulated exocytosis, such a secretory function in the vas deferens could be postulated. Although this defect would account for a decrease in cross progeny, we do observe a coincident decrease in sperm being transferred. This observation means that even if there is a defect in seminal release, there must be some feedback to the gonad to tell it to stop the continuation of transfer. In either case, UNC-18 is necessary for the proper execution of this step.

## Discussion

Understanding how genes function within neural circuits and networks to coordinate a behavioral output is a central focus in neurogenetics. We are using *C. elegans *mating behavior as a model to address how genes control the ability of an animal to execute a stereotyped behavior. During *C. elegans *mating behavior, the male must perceive sensory cues, integrate and process information, and generate the appropriate motor responses in order to successfully mate. Here we have defined the motor outputs (sub-steps), one of the molecules (UNC-18), and parts of the circuitry (site of action) involved in *C. elegans *male sperm transfer, the last step in mating behavior.

Our results indicate that there are at least four sub-steps in the sperm-transfer behavior in *C. elegans*: initiation, release, continued transfer, and cessation. Unlike the other steps of mating behavior, which occur almost instantaneously upon stimulation, the sub-steps of sperm transfer occur over a 90-second period. This observation suggests that a slower acting signal-transduction pathway(s) may be involved in aspects of this behavior.

To gain insight into genetic control of the individual sub-steps of sperm-transfer behavior, we screened for mutants in this process. In a screen for sperm-transfer-defective mutants, we isolated two mutants, *sy671*, defective in sperm-transfer initiation, and *sy672*, defective in the continued transfer of sperm. We cloned and characterized *sy671 *and determined it to be an allele of *unc-18*. UNC-18 is a critical component of the vesicle exocytosis machinery. We infer that the sperm-transfer defects of *unc-18(sy671) *result from a failure in exocytosis. *unc-18(sy671) *has mild locomotor defects, while most other alleles of *unc-18 *have severe movement defects.

We used *unc-18(sy671) *as a tool to further define the circuitry necessary for initiation of sperm-transfer behavior by determining in which tissue/cells UNC-18 acts during this process. We have shown that neuronal expression of *unc-18 *in the ventral nerve cord is required for initiation. Through a combination of transgene and mutant analyses, the input from the ventral-nerve cord is possibly from the male-specific CA motor neurons. Also possible, and not ruled out by our experiments, is that the B-type motor neurons are responsible for the initiation of sperm transfer.

How is the male's ventral nerve cord communicating with the gonad to initiate sperm transfer? Initially it was reported that there was no innervation of the *C. elegans *male gonad. However, reconstruction of the male nervous system has shown neuronal connections to the gonad, for example, through the SPC spicule motor neurons [63]. The connections of the male-specific CA motor neurons are not yet reconstructed, and it will be interesting to see if they, or the B-type ventral-cord motor neurons, innervate the gonad. In *Drosophila melanogaster*, the release of sperm and seminal fluid has been shown to be controlled by a small group of male-specific cholinergic abdominal neurons that innervate male internal reproductive organs [64]. It will be interesting to see if *C. elegans *parallels the configuration in *Drosophila*.

Although restoration of neuron-specific UNC-18 function was able to rescue the initiation defect in *sy671 *males, we determined that animals transferred very few sperm, appearing to initiate, but not continue transfer. Through a series of experiments, we were able to show that non-neuronal expression of *unc-18 *in the male gonad is required for correct continued transfer. Non-neuronal expression of *unc-18 *had not been previously reported in *C. elegans*; however, in other systems, the UNC-18 homologue, Munc-18, is known to function in non-neuronal cells such as the pancreatic β-cells and also chromaffin cells [20-23]. In these cells, Munc-18 is involved in the exocytosis of dense-core vesicles that mediate the release of hormones and possibly other humoral factors. Molecules potentially involved in non-neuronal exocytosis, in particular *unc-64 *(syntaxin), have been described as expressed in non-neuronal tissue in *C. elegans *[41].

The mechanism underlying the ability of gonadally expressed UNC-18 to rescue the continuation of transfer remains to be explored. Garcia et al found that the male somatic gonad, the germ line, and the connection between the vas deferens (somatic gonad) and the cloaca helps to sustain prolonged spicule protraction [15]. Because sustained spicule protraction is necessary for proper continuation and cessation of sperm-transfer behavior, the coordination between sperm transfer and spicule protraction suggests that multiple sensory inputs and feedback are necessary for the proper execution of this behavior, especially from the gonad. Exploring these mechanisms in the future will enhance our understanding of the regulation of these behavioral outputs.

## Conclusion

Our work provides further understanding of how sperm transfer in *C. elegans *is achieved. We have separated the behavior of sperm transfer into four sub-steps: initiation, release, continued transfer, and cessation. In a genetic screen, we isolated an allele of *unc-18 *defective for the initiation sub-step of sperm transfer *(sy671)*, and we determined that the site of action for UNC-18 in the initiation of transfer was in the ventral-cord motor neurons, most likely the male-specific CA neurons. In addition, we have also assigned a role for UNC-18 in the gonad for the proper completion of another sub-step of sperm transfer, the continuation of transfer. We have shown not only that UNC-18 is required in two distinct tissue types for two sub-steps of sperm-transfer behavior (initiation and continued transfer), but also that the SPV neurons that inhibit premature sperm transfer act either upstream or in parallel to the requirement for *unc-18 *in initiation. Further analysis of sperm transfer will hopefully provide novel information about the genetic control of behavior. Because many parasitic nematodes require sperm transfer for reproduction [65], the sub-steps of sperm transfer are potential targets for new antehelminthics.

## Methods

### Strains

*C. elegans *strains were cultured at 20°C or 15°C using standard protocols [66]. The wild-type reference strain, PS3696, contains *plg-1(e2001) *on LGIII [27] and *him-5(e1490) *on LGV [67] and was derived from CB4855 by backcrossing more than 10 times to *him-5(e1490)*. Additional alleles used were: tra-2(q276) LGII, *pha-1(e2123) *[68]*, lin-39(n1880) *[60]*, lin-39(n1760) *[60] LGIII; *unc-31(e169)*[66] LGIV and *egl-15(n484) *[69], dpy-6(e14) [66], unc-18(b403) [31], *lon-2(e678) *[66] LGX. Integrated (*Is*) GFP fusions or extrachromosomal (*Ex*) GFP arrays were: *nIs133[pkd-2::GFP] *(H. Schwartz and H. R. Horvitz, personal communication); *syEx302[(tph-1::GFP] *(Y. Hajdu-Cronin and P. W. Sternberg, unpublished results); *syIs33[gpa-1::GFP] *(L. Jiang and P. W. Sternberg, unpublished results). The following strains are from this work: PS4218 (*him-5(e1490); unc-18(sy671)*), PS4219 (*plg-1(e2001); him-5(e1490); sy672*), PS5107 (*pha-1(e2123); him-5(e1490); syEx783[unc-18::YFP]*), PS4894 (*him-5(e1490); unc-18(sy671); syEx694[unc-18::UNC-18::YFP]*), PS4902 (*him-5(e1490); unc-18(sy671); syEx697[int2itr-1::UNC-18::CFP]*), PS5081 (*him-5(e1490); unc-18(sy671); syEx784[gpa-1::UNC-18::YFP]*), PS4900 (*him-5(e1490); unc-18(sy671); syEx695[unc-119::UNC-18::YFP]*), PS4899 (*him-5(e1490); unc-18(sy671); syEx691[myo-3::UNC-18::YFP]*), PS5125 (*him-5(e1490); unc-18(sy671); syEx785[aex-3::UNC-18::YFP]*), PS5124 (*him-5(e1490); unc-18(sy671); syEx786[unc-25::UNC-18::YFP]*), PS5122 (*him-5(e1490); unc-18(sy671); syEx800[unc-4::UNC-18::YFP]*), PS5123 (*him-5(e1490); unc-18(sy671); syEx787[unc-17::UNC-18::YFP]*), PS5120 (*him-5(e1490); unc-18(sy671); syEx788[cho-1::UNC-18::YFP]*), PS4901 (*him-5(e1490); unc-18(sy671); syEx696[acr-5::UNC-18::YFP]*), PS5073 (*him-5(e1490); unc-18(sy671); syEx789[acr-2::UNC-18::YFP]*), PS5077 (*pha-1(e2123); him-5(e1490); syEx790[acr-5::DsRed2]*), PS5076 (*pha-1(e2123); him-5(e1490); syEx791[acr-5::YFP]*), PS5078 (*pha-1(e2123); him-5(e1490); syEx792[acr-5::CFP]*), PS5072 (*pha-1(e2123); him-5(e1490); syEx793[acr-5::GFP]*), PS5075 (*pha-1(e2123); him-5(e1490); syEx794[acr-2::YFP]*), PS5080 *lin-39 (n1880); him-5(e1490); syEx795[acr-5::GFP]*).

### *unc-18 *transheterozyote strain construction

To create heteroallelic males for the X-linked *unc-18 *locus (*unc-18(sy671) *in *trans *to *unc-18(b403)*) we created the strain in a *tra-2 *background in which XX animals are males [70]**. **We used the *tra-2(q276) *allele, as these males are capable of mating. We crossed *tra-2(q276) *males with *unc-18(sy671) dpy-6(e14) *hermaphrodites, selected non-Dpy non-Unc cross progeny, and allowed them to self-cross. Non-Dpy males from this generation were either *tra-2(q276); +/+ *or *tra-2(q276); unc-18(sy671) dpy-6(e14)/+*. These individual males were crossed with *unc-18(b403) *hermaphrodites, cross progeny from multiple individual lines were selected, and individuals allowed to self-fertilize. Motile males that were non-Dpy were assayed from plates that segregated DpyUnc and Unc. These males are *tra-2 *(*q276); unc-18(sy671) dpy 6 (e14)/unc-18(b403)*. The *tra-2(q276) *control males were extremely poor at the execution of mating behavior, with the majority of them never tonically inserting their spicules during the assay period. Among those that did insert their spicules, 12 of 13 *tra-2(q276) *animals were able to initiate transfer compared with 0 of 16 for the *unc-18 *transheterozygote.

### Laser ablation

We used standard laser-ablation protocols to kill the B.β cell in the male tail [71]. Staged L3 males were mounted, ablated, and allowed to recover and develop into young adults before assay. For sham-operated males, we kept animals on agar pads with the same concentration of sodium azide as the operated males.

### Isolation of mating-behavior mutants

To isolate mutants, we mutagenized PS3696 hermaphrodites using ethyl methanesulfonate (EMS) [66], and placed P0 animals on individual plates. From each plate, three F1 animals were pooled on a new plate, and 10 individual animals from the F2 generation were placed onto individual plates. The F3 populations were scored for mating plugs. The *him-5 *mutation increases the incidence of males in the population from <1% to 33%, and the *plg-1 *allele causes males to secrete a mucus plug over the vulva after sperm transfer [27][67].

Using this approach, approximately 95% of the initial F3 populations were eliminated as either plug-positive or sterile, leaving 5% enriched for mating-defective mutants. These 'plug-less' lines were subsequently assayed by observing mating behavior directly, and assigned a corresponding defect. From the 1400 haploid genomes screened, we isolated two sperm-transfer-defective mutants, *sy671 *and *sy672*. The *sy671 *animals were defective in initiation, while *sy672 *animals were defective for continued transfer. *sy671 *animals were out-crossed at least three times with N2 strains before subsequent assays.

### Mapping and cloning of *sy671*

For SNP mapping we used the Hawaiian CB4856 strain as the source of the polymorphisms [29]. To analyze the SNPs, we used the recommended primer pairs [72] to amplify the genomic regions of the recombinants for the following markers:

• Chromosome I: ZC123:21628, Y71G12A:7551, W03D8:34384, F57C9:23691, T07D10:11633

• Chromosome II: T01D1:3656, T13C2:22236, Y51B9A:7379, Y38F1A:41752

• Chromosome III: H06104:15629, T28D6:5428

• Chromosome IV: C45G7:33902, ZK792:2021, W02A2:25818, Y41E3:104573

• Chromosome V: B0213:10033, T25E12:1056

• Chromosome X: ZC449:15947, K10C2:8278, C01C10:13818, F26A10:1544, ZK154:6193, C54D2:9060, F45E1:9628, F11A1:9511, R04E5:10898, C05E7:28741.

In addition, the region between ZK154 and F26A10 on chromosome X did not have any SNP data; therefore, we sequenced three intergenic 1.5-kb regions in this interval from the CB4856 strain to find polymorphisms. Two of the three sequenced fragments revealed the new SNP markers C47C12:6306 and F27D9:6408. Primers and information about these markers are detailed below.

• C47C12:6306: amplification with the following primers, C47C12:6306A (5'-ATTTCTTGCTCTGCTTCAACATCC-3') and C47C12:6306B (5'-GAACATGACAGTAAGCAATCACG-3') amplifies an 820-bp fragment. Digestion with *Nde*II (GATC) gives two fragments in N2 (93 and 727 bp) and three fragments in CB4856 (93, 534, and 193 bp) as there is an A (N2) to T (CB4856) change (underlined) in the following sequence: TCTTTCACTTTCCAATGTGAACTGATATATATTGATGGGGT.

• F27D9:6408: amplification with the following primers, F27D9:6408E (5'-GAGACCTCCTTGCTCAATGACC-3') and F27D9:6408F (5'-ACGTGACAATATACGAGATTGAGC-3') amplifies a 786-bp fragment. The change is A (N2) to T (CB4856) at nucleotide 626 in the following sequence: AGTATAGAATTCCACGTGACAGAAAAATCTGTATTATTGTA. As no restriction endonuclease exists that can resolve this change, sequencing is required in order to genotype.

Our initial mapping populations included 18 mutant and six wild-type F3 populations (total of 48 chromosomes analyzed) from a cross between the Hawaii strain (CB4856) and *sy671*. These populations were used to map *sy671 *to the X chromosome, between the markers ZC449 and F11A1. The numbers adjacent to the marker shown below are the number of recombinant chromosomes/total analyzed for that marker:

• Chromosome I: ZC123 (22/32), Y71G12A (18/48), W03D8 (15/48), F57C9 (12/36), T07D10 (13/34)

• Chromosome II: T01D1 (14/30), T13C2 (25/36), Y51B9A (22/36), Y38F1A (20/34)

• Chromosome III: H06104 (17/36), T28D6 (16/36)

• Chromosome IV: C45G7 (8/36), ZK792 (14/38), W02A2 (10/48), Y41E3 (8/36)

• Chromosome V: B0213 (14/48), T25E12 (13/48)

• Chromosome X: ZC449 (4/48), F45E1 (0/48), R04E5 (0/22), C05E7 (11/36).

For generation of additional recombination events in our interval of interest, we generated the triple mutant *lon-2(e678) unc-18(sy671) egl-15(n484) *for further SNP mapping. To help avoid potential modifiers of mating behavior from the Hawaii strain, we intragressed the CB4856 sequence into the *lon-2 egl-15 *interval of N2 by crossing CB4856 with the *lon-2 egl-15 *double mutant and selecting a non-Lon non-Egl F3 population (three times). The intragressed CB4856 strain was checked with internal SNP markers and then crossed with *lon-2 (e678) unc-18(sy671) egl-15(n484)*. In total, 99 Lon non-Egl recombinants were selected, made homozygous, and used for mapping *sy671 *to a five-cosmid interval.

### Mutant rescue

All cosmids, including the rescuing cosmid F27D9, were injected into *unc-18(sy671) *at 20 ng/μl with *myo-2::GFP *(5 ng/μl) as a co-injection marker and pBSKS(175 ng/μl) as a carrier. In addition, a rescuing 7.2-kb PCR fragment was amplified using the following primers F27D9P5F (5'-TCGTGACGATCTAGAAGTGGCATTCC-3') and F27D9P5R (5'-TTGGCTTCTCAACGTGGAATGACTGG-3') and purified using the QiaQuick PCR purification kit. The PCR fragment was injected at the same concentration as the cosmid. Transgenic animals were recognized by *myo-2::GFP *expression in the pharynx. Rescue was also achieved using a 5-kb fragment containing 2.6 kb of 5' upstream promoter sequence and the entire genomic region of *unc-18*.

### PCR and sequencing

Three overlapping fragments covering a 2.6-kb genomic DNA region containing the entire *unc-18 *coding region were amplified from *sy671 *mutant DNA using PCR with the following primer pairs: F27d9.1A (5'-TTTCCGTCTCATGTTCTTCGCTCC-3') and F27d9.1B (5'-TTCTCTCAAAGTCAGCACGGTACC-3'), F27d9.1C (5'-TGCGCAACACTTGGAGAATATCC-3' and F27d9.1D (5'-TGATTGTTTCCTTATCAGCCATGG-3'), and F27d9.1E (5'-GATGGTGCCACTTTTGATTGACC-3') and F27d9.1F (5'-CTGGTGGGAGAATAAGAAAATTCC-3'). The PCR products from two independent reactions for each pair were separately purified using a QIAquick PCR purification kit and sequenced directly. The G→A nucleotide change causing the sperm-transfer phenotype in *sy671 *was found at nucleotide 346 using the E and F primer set. The mutation is in the following sequence: GCAAGTTTACCAATCTTCCC**G**CTGGGTTCCAGTTATCAAGG. As this mutation creates an additional site for restriction endonuclease digestion using *TspR*I, the mutation was rechecked by *TspR*I digestion of the PCR product. The Arg476His change is numbered according to UNC-18 isoform A. Currently there are three described isoforms A, B, and C [73] and this change affects isoforms A and C (WormBase WS130).

### Observations of mating behavior

We observed mating behavior with a Zeiss M2-Bio dissecting microscope, up to 660× magnification. The mating behavior of mutant or control males was observed with sluggish *unc-31 *adult hermaphrodites. All males were isolated from sibling hermaphrodites at the L4 stage and were kept on fresh plates in groups before observation [10,15]. For the mating assay, a virgin adult male (18–24 hrs post L4 lethargus) was placed on a 10–20 mm bacterial lawn with multiple *unc-31 *hermaphrodite adults. The hermaphrodites used were between 3 and 4 days post L4, as these are partners with which spicule insertion occurs almost instantaneously [15], facilitating analysis. After tonic spicule insertion, a stopwatch was started. Time points were taken when the sperm were released into the hermaphrodite (release) and when the spicules were retracted after sperm transfer (cessation). Because the time difference between initiation and release is generally of the order of 1–2 seconds, a Sony DFW-V500 color digital camera was used to record this behavioral interval from a separate group of animals, as a stopwatch was not suitable. These videos were later analyzed using iMovie (Apple).

When testing animals specifically for sperm-transfer initiation, animals were assayed until the first tonic spicule insertion and transfer was noted. If an animal did not insert their spicules within 10 minutes, that animal was not scored.

We observed that older *sy671*males (48 hours post-L4) would initiate sperm transfer more often than the 24-hour post-L4 *sy671 *males used in our assays. Although these males initiate more often, they will not transfer as many sperm as wild type.

### Continuation assays

L4 *unc-31 *hermaphrodites were staged and used 3.5 days later for mating with isolated 18–24-hour post L4 virgin adult males. After an individual mating event, the hermaphrodite was moved to a fresh plate. The progeny sired (both cross and self) were tallied to determine the percentage of cross progeny. Percentage of cross progeny is the number of cross progeny divided by the total number of progeny.

### Extrachromosomal array loss

Progeny of *unc-18(sy671) *animals carrying both *Ex[Punc119::unc-18::yfp] *and *Ex[Pint2itr-::unc-18::cfp] *transgenes were scored as L4 males to determine if either YPF or CFP fluorescence was lacking in these animals. Animals completely lacking either *Punc119::unc-18::yfp *or *Pint2itr-::unc-18::cfp *were identified using a Zeiss M2Bio stereofluorescence microscope with the appropriate filter. These animals were then assayed for mating behavior, and then loss of fluorescence confirmed using conventional fluorescence microscopy (Zeiss Axioskop) after assay.

### Sperm isolation and *in vitro *activation

Individual males were placed in a 7-μl drop of sperm media [74] containing 10 mg/ml PVP and 200 μl/ml pronase [62]. Dissections to release sperm were accomplished by cutting in front of the tail with a hypodermic needle [74], and released spermatids were observed as they became activated to form spermatozoa within 5 minutes on a Zeiss Axioskop with DIC optics.

### Molecular biology

For *unc-18 *reporter construction (*unc-18::YFP*), the *unc-18 *promoter was amplified by PCR using N2 genomic DNA as the template with the primers 5'-AAACTGCAGTGAAGGACAATGAACTAGAGGGAC-3' and 5'-CGCGGATCCGTGCCCAACGATTTGTTTGAGTGAC-3'. The PCR product was digested with *Bam*HI and *Pst*I, and ligated into *Bam*HI/PstI-digested pSX95.77 (courtesy of S. Xu). This plasmid contains the *yfp *coding region followed by the *unc-54 *3' UTR from pPD136.64 (a gift of A. Fire). *unc-18::YFP *was injected at 75 ng/μl with pBX-1 [75] (100 ng/μl) and pBSKS (25 ng/μl) into *pha-1(e2123ts); him-5(e1490) *animals [75,76]. Males from three independent transgenic lines carrying the extrachromosomal array were analyzed for *unc-18::YFP *expression.

The *unc-18 *rescue construct (*unc-18::UNC-18::YFP*) was made using the following primers, 5'-AAAACTGCAGGTGACGATCTAGAAGTGGCATTCC-3' and 5'-CGCGGATCCTATGTCACGCGGTTTGTTCAGG-3'. We amplified by PCR a 5-kb fragment that contained a 2.4 kb sequence upstream of the *unc-18 *start codon through the entire coding region, except the stop codon, using cosmid F27D9 DNA as a template. This fragment was digested with *Pst*I and *Bam*HI, and was cloned in-frame into the corresponding sites of pSX95.77, creating a YFP-tagged form of UNC-18 (*unc-18::UNC-18::YFP*). This plasmid was injected into *unc-18(sy671) *at 100 ng/μl with *myo-2::GFP *(5 ng/μl) and pBSKS (95 ng/μl).

For site-of-action studies, a promoterless 4.2-kb fragment from the ATG of *unc-18 *through the end of *unc-54 *was amplified by PCR from the *unc-18::UNC-18::YFP *plasmid using the following primers: 5'-AAAACTGCAGAAAAATGTCACTCAAACAAATCGTTGG-3' and 5'-CCCAAGCTTTGATGCGGTATTTTCTCCTTACG-3'. The PCR product was digested with *Hin*dIII and *Pst*I, and ligated into *Hin*dIII/*Pst*I-digested pBSKS, creating pSOAunc-18.

To create *myo-3::UNC-18::YFP *we amplified by PCR the *myo-3 *promoter region [77] using the following primers: 5'-ataagaatGCGGCCGCTTGATAAGGCTGCAACAAAGATCAGG-3' and 5'-ATAGTTTAGCGGCCGCTTCTAGATGGATCTAGTGGTCGTGG-3'. The PCR product was digested with *Not*I, ligated into *Not*I-digested pSOAunc-18, and the orientation checked.

The following were constructed as for *myo-3::UNC-18::YFP *(above), except that the primers for amplification of the appropriate promoter regions were unique:

• unc-17 [52]: 5'-ataagaatGCGGCCGCCATTACGTTTCACATTGTCAGAAGCCAAACG-3' and 5'-ATAGTTTAGCGGCCGCTAACAGAGCCGTGAGACCCATTGTATGG-3'

• gpa-1 (Jiang LI and Sternberg PW unpublished observations): 5'-ATAAGAATGCGGCCGCTGCTTGAAGAACACAGTATCTACG-3' and 5'-ATAGTTTAGCGGCCGCCTGAAGTCTTCGAATAAATGACATTG-3'.

To create the *unc-119::UNC-18::YFP *plasmid, we amplified by PCR the *unc-119 *promoter region [43] using the following primers: 5'-ATAAGAATGCGGCCGCCTATTCCTAGACGATTATTGGTTCC-3' and 5'-CCAATGCATTGGTTCTGCAGATATGCTGTTGTAGCTGAAAATTTTGG-3'. The PCR product was digested with *Not*I and *Pst*I, and ligated into *Not*I/*Pst*I-digested pSOAunc-18.

The following were constructed as for *unc-119::UNC-18::YFP *(above), except the primers for amplification of the appropriate promoter regions were unique:

• acr-2 [54]: 5'-ATAAGAATGCGGCCGCCAAGTGTTGCAGCGACATTATTCAGG-3' and 5' '-CCAATGCATTGGTTCTGCAGAAAACGGCGTCCTTCCTGTGAAGG-3'

• acr-5 [57]: 5'-ATAAGAATGCGGCCGCAATGATTGGCGAGATGCTCATGAAGGTCG-3' and 5' CCAATGCATTGGTTCTGCAGGCTGAAAATTGTTTTTAAAGCATTGAAACTGG-3'

• aex-3 [45]: 5'-ATAAGAATGCGGCCGCTGTTCCAAAATGACCAAGTATC-3' and 5'-CCAATGCATTGGTTCTGCAGATTAGGATAGGTACATTGGTGC-3'

• int2itr-1: 5'-ATAAGAATGCGGCCGCTGAGGAACAAATTATGAGGAAATCCAGG-3' and 5'-CCAATGCATTGGTTCTGCAGGAAGCAATGGATGACTGTGCTTTTAGC-3'

• unc-25 [51]: 5'-ATAAGAATGCGGCCGCTTCATAAGACGCCAGGCGAGCACTCG-3' and 5'-CCAATGCATTGGTTCTGCAGGCGGTGAACTGAGCTTTTCCCTATTCC-3'

• unc-4 [56]: 5'-ATAAGAATGCGGCCGCGTGAAATACTCAATCAATCACTCTGG-3' and 5'-CCAATGCATTGGTTCTGCAGTCACTTTTTGGAAGAAGAAGATCCTC-3'.

The *unc-119 *reporter (*unc-119::YFP*) was created by digesting *unc-119::UNC-18::YFP *with *Pst*I and *Sma*I to drop out the *unc-18 *coding region, followed by removal of the 3' overhang with T4 polymerase and re-ligation.

*acr-5::YFP; acr-2::YFP; aex-3::YFP*, and *int2itr-1::YFP *were all constructed by excising the *unc-18 *region from the appropriate construct above with *PstI *and *SmaI*, following which the resulting vector fragment was blunt-ended with T4 polymerase and the vector relegated. a*cr-5::GFP *was constructed by swapping the *yfp *from *acr-5::YFP *with a nuclear localized version of *gfp *from pPD95.70 (a gift of A. Fire).

The *acr-5*::*DsRed2 *reporter was constructed as follows. The *acr-5 *promoter was removed by linearization of *acr-5::UNC-18::YFP *by digestion with *Not*I, followed by Klenow treatment and subsequent digestion with *Pst*I, creating a blunt/*Pst*I promoter fragment. This fragment was ligated into a vector containing *DsRed2 *followed by the *unc-54 *3' UTR (PSX77-*DsRed2 *courtesy of S. Xu) that was prepared by digestion with *Hin*dIII, Klenow-treated, and subsequently cut with *Sse*8387 I.

Because the promoter region of *cho-1 *[53] was not stable in pSOAunc-18, we excised the *Bss*HII fragment containing the ATG of *unc-18 *through the end of the *unc-54 *3' UTR from pSOAunc-18, treated it with T4 polymerase, and blunt-end cloned it into the *Eco*RV site in pBR322. We then PCR amplified the *cho-1 *promoter with the primers 5'-TCCCCGCGGATACTTCAATTATTCCCGTCTTACCAGG-3' and 5' '-TCCCCGCGGTAAGATAGAGGAACTCGCAAGATGTCG-3', digested with *Sac*II, and cloned into the *Sac*II site carried over to pBR322 and checked the orientation.

To make *int2itr-1::UNC-18::CFP*, we digested *int2itr-1::UNC-18::YFP *with *Not*I/*Sma*I to excise the *int2itr-1 *promoter followed by the *unc-18 *coding region (leaving the *yfp *and *unc-54 *3' UTR sequence) and ligated in frame into pBSKS containing the *cfp *coding region followed by the *unc-54 *3' UTR.

### High-resolution light microscopy

Cell anatomy was observed with Nomarski optics, and GFP expression was analyzed by conventional fluorescence microscopy (Zeiss Axioskop) using a Chroma Technology High Q GFP long-pass filter set (450 nm excitation, 505 nm emission). CFP and YFP were visualized using a Chroma Technology CFP filter set (31044v2; exciter D436/20, emitter D480/40, beamsplitter 455dclp) and a YFP set (41029; exciter HQ 500/20, emitter HQ520lp, beamsplitter Q515lp). Photographs were taken with a digital camera using Improvision Openlab software to obtain images, and images processed using Adobe Photoshop version 7.0.

## Authors' contributions

GS, AJW and PWS conceived the study. GS, AJW, JYT, and SG all contributed to the genetic screening and the initial characterization of isolated male mating-behavior mutants. GS performed laser ablations, genetic manipulations, and behavioral assays on transformants. JYT was involved in the behavioral assays on transformants. GS drafted the manuscript, with AJW, JYT, SG, and PWS revising it critically for important intellectual content. All authors read and approved the final manuscript.

## Supplementary Material

Additional file 1 Initiation. The initiation step of sperm transfer Click here for file

Additional file 2 Release and continued transfer. The release and continued transfer steps of sperm transfer Click here for file

Additional file 3 Cessation. The cessation step of sperm transfer Click here for file

Additional file 4 Results from individual lines complied for Table 1 Click here for file

Additional file 5 Results from individual lines complied for Table 3 Click here for file

Additional file 6 Neuronal and non-neuronal expression of UNC-18 are necessary for sperm transfer. Click here for file
