# Supplementary material for: Initiation of male sperm-transfer behavior in Caenorhabditis elegans requires input from the ventral nerve cord
Source: BMC Biol. 2006 Aug 15;4:26. doi: 10.1186/1741-7007-4-26 (PMC1564418; doi:10.1186/1741-7007-4-26)
Supplement: Additional file 4 — Results from individual lines complied for Table 1 [file 1741-7007-4-26-S4.doc]

Results from individual lines complied for Table 1

| Genotype | Initiate/  total | Individual lines ^a^ - Initiate/total | | | | | | |
| --- | --- | --- | --- | --- | --- | --- | --- | --- |
|  |  | 1 | 2 | 3 | 4 | 5 | 6 | 7 |
| *unc-18(sy671); Ex[Punc18::unc-18::yfp]* | 37/42^b^ | 16/18 | 11/12 | 10/12^d^ |  |  |  |  |
| *unc-18(e81); Ex[Punc18::unc-18::yfp]* | 27/30 ^b^ | 12/13 | 7/7 | 8/10 |  |  |  |  |
| *unc-18(sy671); Ex[Promoterless::unc-18::yfp]* | 0/26^c^ | 0/16 | 0/10 |  |  |  |  |  |
| *unc-18(sy671); Ex[Pint2itr-::unc-18::yfp* | 0/84 ^c^ | 0/17 | 0/17 | 0/16 | 0/17 | 0/17 |  |  |
| *unc-18(sy671); Ex[gpa-1::unc-18::yfp]* | 3/93 ^c^ | 0/17 | 0/16 | 2/22 | 0/19 | 1/19 |  |  |
| *unc-18(sy671); Ex[Punc119::unc-18::yfp* | 70/106 ^b^ | 18/22^d^ | 12/14 | 6/16 | 12/15 | 7/11 | 6/14 | 9/14 |
| *unc-18(sy671); Ex[myo-3::unc-18::yfp* | 0/87 ^c^ | 0/14 | 0/12 | 0/13 | 0/12 | 0/12 | 0/12 | 0/12 |
| *unc-18(sy671); Ex[aex-3::unc-18::yfp]* | 29/34 ^b^ | 10/12^d^ | 9/11 | 10/11 |  |  |  |  |

^a^ Of the multiple transgenic lines created for each construct, only those that had relatively consistent YFP expression were analyzed. In addition, animals from each line that were assayed were checked at the gross level for proper YFP expression.

^b^ All individual lines were significantly different from *unc-18(sy671)* (*p*<0.05) using ANOVA with

aTukey-Kramer multiple comparisons test (InStat3 software). *unc-18(sy671)* data from Table 1.

^c^ All individual lines were not significantly different from *unc-18(sy671)* (*p*>0.05) using ANOVA

with aTukey-Kramer multiple comparisons test (InStat3 software). *unc-18(sy671)* data from

Table 1.

^d^ Line used in subsequent assays.
