# Supplementary material for: Initiation of male sperm-transfer behavior in Caenorhabditis elegans requires input from the ventral nerve cord
Source: BMC Biol. 2006 Aug 15;4:26. doi: 10.1186/1741-7007-4-26 (PMC1564418; doi:10.1186/1741-7007-4-26)
Supplement: Additional file 6 — Neuronal and non-neuronal expression of UNC-18 are necessary for sperm transfer. [file 1741-7007-4-26-S6.doc]

**Neuronal and non-neuronal expression of UNC-18 are necessary for sperm transfer.**

| Genotype | Transgene expression | | **Init.^a^** | **Cont.^b^** | **% Cross- progeny^c^** | **n^d^** | ***p* value ^f^** |
| --- | --- | --- | --- | --- | --- | --- | --- |
|  | **Gonad** | **Neuron** |  |  |  |  |  |
| Wild-type ^g^ | **None** | **None** | **Yes** | **Yes** | **74.4 ± 7.1** | **29** |  |
|  |  |  |  |  |  |  |  |
| ***unc-18*^h^** | **None** | **None** | **No** | **N/A** | **0 ± 0** | **16** |  |
|  |  |  |  |  |  |  |  |
| ***unc-18; Ex[******unc18::UNC-18::YFP]*** | **Yes** | Yes | **Yes** | **Yes** | **71.1 ± 8.5** | **7** | **0.41 vs. wild-type** |
|  |  |  |  |  |  |  |  |
| ***unc-18(e81); Ex[unc18::UNC-18::YFP]*** | **Yes** | Yes | **Yes** | **Yes** | **67.6 ± 12.9** | **8** | **0.13 vs. wild-type** |
|  |  |  |  |  |  |  |  |
| ***unc-18; Ex[unc-119::UNC-18::YFP]*** | **No** | **Yes** | **Yes** | **No** | **21.6 ± 24.7** | **16** | **<0.0001 vs. wild-type,**  **0.009 vs.** ***unc-18(sy671)*  and 0.2 vs. *unc-18(sy671);***  ***Ex[unc-119::UNC-18::YFP]***  **Lose *Ex[int2itr-1::UNC-18::CFP]*^e^** |
|  |  |  |  |  |  |  |  |
| ***unc-18; Ex[unc-119::UNC-18::YFP]; Ex[int2itr-1::UNC-18::CFP]*** | **Yes** | **Yes** | **Yes** | **Yes** | **69.3 ± 12.6** | **8** | **0.46 vs. wild-type** |
|  |  |  |  |  |  |  |  |
| ***unc-18; Ex[unc-119::UNC-18::YFP];* Lose *Ex[int2itr-1::UNC-18::CFP]*^e^** | **Lost** | **Yes** | **Yes** | **No** | **10.2 ± 19.7** | **13** | **<0.0001 vs. wild-type** |
|  |  |  |  |  |  |  |  |
| ***unc-18; Ex[int2itr-1::UNC-18::CFP];* Lose Ex*[unc-119::UNC-18::YFP*]^e^** | **Yes** | **Lost** | **No** | **N/A** | **6.7 ± 18.9** | **13** | **<0.0001 vs. wild-type**  **0.4 vs. *unc-18(sy671)*** |
|  |  |  |  |  |  |  |  |
| ***unc-18; Ex[acr-2::UNC-18::YFP]*** | **No** | **Yes** | **Yes** | **No** | **15.9 ± 21.1** | **14** | **<0.0001 vs. wild-type**  **0.59 vs. *unc-18; Ex[unc-119::UNC-18::YFP]*** |
|  |  |  |  |  |  |  |  |
| ***unc-18; Ex[acr-5::UNC-18::YFP]*** | **No** | **Yes** | **Yes** | **No^i^** | **21.7 ± 30** | **17** | **<0.0001 vs. wild-type**  **0.78 vs. *unc-18; Ex[unc-119::UNC-18::YFP]*** |
|  |  |  |  |  |  |  |  |
| ***unc-18; Ex[aex-3::UNC-18::YFP]*** | **Yes** | **Yes** | **Yes** | **Yes** | **59.8 ± 36** | **16** | **0.66 vs. wild-type**  **0.003 vs. *unc-18; Ex[unc-119::UNC-18::YFP]*** |
| **^a^  Initiation was scored as positive when sperm exit the seminal vesicle, regardless of amount.**  **^b^ Continuation was scored as positive when it was observed that the animals transferred what appeared to be a wild-type amount of sperm, later confirmed by % cross-progeny.**  **^c^ Mean number of cross-progeny and standard deviation.**  **^d^ Number of animals assayed.**  **^e^ indicates that this extrachromosomal array was lost during meiosis and the animal therefore only carries one type of extrachromosomal array. The parental strain was *plg-1(e2001d;him-5(e1490);unc-18(sy671) Ex[unc-119::UNC-18::YFP] Ex[int2itr-1::UNC-18::CFP].***  **^f^ *p* values were calculated using the Mann-Whitney test.**  **^g^ Wild-type is strain PS3696, *plg-1(e2001)*;*him-5(e1490).***  **^h^ *unc-18* allele used is *sy671* unless specified**  **^i^ Three animals transferred wild-type amounts** | | | | | | |  |
